# Artificial Intelligence and Public Health: Evaluating ChatGPT Responses to Vaccination Myths and Misconceptions

**DOI:** 10.3390/vaccines11071217

**Published:** 2023-07-07

**Authors:** Giovanna Deiana, Marco Dettori, Antonella Arghittu, Antonio Azara, Giovanni Gabutti, Paolo Castiglia

**Affiliations:** 1 Department of Biomedical Sciences, University of Sassari, 07100 Sassari, Italy; giovanna.deiana90@gmail.com; 2 Department of Medical, Surgical and Experimental Sciences, University Hospital of Sassari, 07100 Sassari, Italy; azara@uniss.it (A.A.); castigli@uniss.it (P.C.); 3 Department of Medicine, Surgery and Pharmacy, University of Sassari, 07100 Sassari, Italy; aarghittu@uniss.it; 4 Department of Restorative, Pediatric and Preventive Dentistry, University of Bern, 3012 Bern, Switzerland; 5 Working Group “Vaccines and Immunization Policies”, Italian Society of Hygiene, Preventive Medicine and Public Health, 16030 Cogorno, Italy; giovanni.gabutti@unife.it

**Keywords:** ChatGPT, vaccines, immunization, myths and misconceptions, public health, artificial intelligence

## Abstract

Artificial intelligence (AI) tools, such as ChatGPT, are the subject of intense debate regarding their possible applications in contexts such as health care. This study evaluates the Correctness, Clarity, and Exhaustiveness of the answers provided by ChatGPT on the topic of vaccination. The World Health Organization’s 11 “myths and misconceptions” about vaccinations were administered to both the free (GPT-3.5) and paid version (GPT-4.0) of ChatGPT. The AI tool’s responses were evaluated qualitatively and quantitatively, in reference to those myth and misconceptions provided by WHO, independently by two expert Raters. The agreement between the Raters was significant for both versions (*p* of K < 0.05). Overall, ChatGPT responses were easy to understand and 85.4% accurate although one of the questions was misinterpreted. Qualitatively, the GPT-4.0 responses were superior to the GPT-3.5 responses in terms of Correctness, Clarity, and Exhaustiveness (Δ = 5.6%, 17.9%, 9.3%, respectively). The study shows that, if appropriately questioned, AI tools can represent a useful aid in the health care field. However, when consulted by non-expert users, without the support of expert medical advice, these tools are not free from the risk of eliciting misleading responses. Moreover, given the existing social divide in information access, the improved accuracy of answers from the paid version raises further ethical issues.

## 1. Introduction

Large Language Models (LLMs) are a type of Artificial Intelligence (AI) designed to reproduce human language processing capabilities. They use deep learning techniques, such as artificial neural networks, and are capable of learning and processing large amounts of language data from various sources [1,2]. With extensive training they can generate highly coherent and realistic text. LLMs analyze patterns and connections within the data they have been trained on and use that knowledge to understand and generate language in various fields such as machine translation and text generation [3,4]. LLMs have become increasingly common over the past decade and have been applied across a variety of sectors, including content marketing, customer services, and numerous business applications [5,6].

Launched on 30 November 2022, ChatGPT, an AI-based LLM developed as a non-profit venture by OpenAI (OpenAI, L.L.C., San Francisco, CA, USA), is an advanced modeling conversational chatbot, a program that can understand and generate responses using a text interface. It has gained widespread popularity in a very short time and its latest version GPT-4.0 was released on 14 March 2023. Two versions are currently available: GPT-3.5, which is free to use and is the fastest version, and GPT-4.0, which must be paid for and is considered the most capable version [7,8].

This conversational system is based on a Generative-Pre-Trained Transformer (GPT) architecture, an LLM with over 175 billion parameters, which can be trained on a broad range of internet sources in multiple languages, including books, articles, and websites [9]. ChatGPT shows considerable proficiency in understanding natural language text and is able to generate highly sophisticated, tailored, human-like responses based on detailed questions related to the context of the input text [10,11]. The LLM in ChatGPT uses supervised fine-tuning modeling, reward model building, proximal policy optimization, and reinforcement learning from human feedback, enabling it to incorporate the complexity of user intentions and respond profitably to various end-user activities, interacting in a conversational manner [12,13].

In the scientific and academic community, the rise of LLMs has generated great interest and inspired an increasingly sophisticated debate about the relative benefits and risks and their ethical implications [14,15]. On the one hand, LLMs can be useful in speaking and writing tasks, helping to increase the efficiency and accuracy of the required output. Moreover, they could be incorporated into teaching and learning, such as mentoring and student assignment assistance, and also into academic writing, where these tools could help researchers optimize the time needed for manuscript preparation [16,17]. On the other hand, concerns have been raised about possible biases based on the datasets used, which may limit their capabilities and could result in factual inaccuracies. Additionally, security issues and the potential for spreading misinformation must be considered, as well as ethical and fair access issues relating to the accessibility of these digital tools with particular reference to the availability of a paid version [18,19].

LLMs have also generated intense interest and debate among healthcare professionals and medical researchers, considering their potential to improve health and patients’ lives [20,21]. In particular, taking into consideration the growing amount of medical data and the complexity of clinical decisions, these tools could theoretically help physicians make timely and informed decisions and improve the overall quality and efficiency of healthcare [22,23]. Reference is made here to “long-distance care”, a concept regarding the use of digital technologies to support the healthcare system in order to make service delivery more effective, streamline the communication between healthcare facilities and citizens, simplify booking systems, and ensure quality healthcare. In particular, in the years of the COVID-19 pandemic, in which the digital transition of the health sector was accelerated, examples of this kind were witnessed with advanced technologies related to AI, tele-medicine, tele-rehabilitation, self-medication, digital health interventions (e-health and m-health), electronic referral and online counseling systems, and systems for monitoring and measuring healthy lifestyle behaviors (e.g., remote monitoring of physical activity and proper nutrition, digital education, and self-medication) [24,25,26].

However, while on the one hand numerous efforts have been made in public health to enable citizens to make informed health choices (e.g., voluntary adherence to vaccination) for example, through digital technologies, on the other hand, the World Wide Web is saturated with data and information, and not all of the information may be accurate. This appears even more worrisome when one considers that, nowadays, technological advances have led to the democratization of knowledge, whereby patients no longer rely solely on healthcare professionals for medical information, but they provide their own health education and information themselves. Monitoring this trend through the study of people’s behaviors and attitudes could be a useful tool to help public health authorities, in guiding vaccination policies, designing new health education, and continuing information interventions aimed at both the general public and responsible cohorts such as health care workers [27,28,29].

This has been evident during the recent COVID-19 pandemic, particularly with regard to vaccinations [30,31]. Indeed, previous evidence has shown that the reliance on online sources of information, which can sometimes provide authoritative answers to complex medical questions, was significantly associated with a greater tendency to vaccine hesitancy and a lower willingness to adhere to vaccination recommendation [32,33]. Moreover, anti-vaccine content on the Web exacerbated the already precarious decision-making process, a dynamic conditioned by the traditional influence of social, cultural, political and religious determinants on vaccine acceptance. Due to a marked decrease in vaccine coverage, this exposes the population to the risk of the reappearance of infectious diseases now under control. Despite the fact that the COVID-19 pandemic has reaffirmed the importance of vaccination as an indispensable tool of primary prevention, the vaccine hesitancy phenomenon continues to affect more than 15 percent of the world’s population, compounded by the recent phenomenon of vaccine fatigue [34,35].

Moreover, in addition to the presence of incorrect information on the Web that can exacerbate vaccine hesitancy, the enormous body of information available online is not equally accessible to the entire population. Nowadays, the digital divide represents a recognized critical aspect of health inequality [36,37].

Given the importance of accurate information regarding vaccines, the study aimed to determine the Correctness, Clarity, and Exhaustiveness of ChatGPT’s responses to misleading questions about vaccines and immunization, in order to: (i) evaluate how these new information tools may be able to provide relevant and correct information with regard to vaccination adherence; (ii) evaluate if GPT-3.5, being free, has significant differences from the more advanced, paid version; and (iii) evaluate whether the use of AI, such as ChatGPT, could help increase health literacy and reduce vaccine hesitancy.

## 2. Materials and Methods

### 2.1. Study Design

The study was based on the answers given by ChatGPT to the list of the 11 questions concerning “Vaccines and immunization: Myths and misconceptions” published on 19 October 2020, taken into consideration alongside those given by the World Health Organization (WHO) (Table 1) [38].

This list, originally written by the U.S. Centers for Disease Control and Prevention, addresses common misconceptions about vaccination that are often cited by concerned parents as reasons to question the wisdom of having their children vaccinated [39]. Thus, the WHO responded to the listed questions, in order to provide a useful information tool for the general population and health professionals charged with carrying out vaccination. In order to assess whether the answers provided by the chatbot were equally accurate, the listed questions were administered in an individual chat by an investigator (G.D.) to both the free (GPT-3.5) and paid (GPT-4.0) versions of ChatGPT.

### 2.2. Quantitative and Qualitative Analysis

ChatGPT responses were independently assessed by two Raters with proven experience in vaccination and health communication topics (P.C. and G.G.), randomly identified as Rater 1 and Rater 2. The Raters were aware of the chatbot version from which the answer was formulated. The responses were evaluated according to predefined scales of accuracy considering three items: Correctness, Clarity, and Exhaustiveness. Each response was rated using a 4-point Likert scale scoring from 1 (strongly disagree) to 4 (strongly agree).

The Raters qualitatively analyzed the responses according to the following determinants: (i) Correctness, in terms of plausibility, coherence, scientific veracity, and evidence; (ii) Clarity, in terms of ease of understanding, appropriateness of vocabulary, conciseness, and logical order; (iii) Exhaustiveness, in terms of the degree of completeness of the answer.

### 2.3. Statistical Analysis

Results were recorded descriptively as mean (±standard deviation; percentage); the percentage was calculated by the formula: (Xob − Xminp)/(Xmaxp − Xminp) × 100, where Xob is “Obtained score”; Xminp is “Minimum score”; and Xmaxp is “Maximum score”. Differences observed in the scores across ChatGPT versions were compared using the Mann–Whitney U test. Inter-observer reliability and overall agreement between Raters were assessed using Cohen’s kappa statistic on all scores. Differences between proportions were tested with the z-test. A statistical significance of *p*-value < 0.05 was set for all analyses. Differences among groups were tested via the Kruskal–Wallis H test. Statistical analyses were performed with STATA 17 (StatsCorp., College Station, TX, USA).

## 3. Results

### 3.1. Quantitative Analysis

Overall, 132 scores were obtained: 11 questions per 3 items per 2 ChatGPT versions per 2 Raters. The scores are listed in Table 2, divided into four groups.

The average answer score for the eleven questions was: 3.18 (±0.80; 79.5%) for GPT-3.5 and 3.61 (±0.65; 90.2%) for GPT-4.0 according to Rater 1; and 3.30 (±0.94; 82.6%) for GPT-3.5 and 3.58 (±0.65; 89.4%) for GPT-4.0 according to Rater 2.

Considering the four groups, Rater 1 assigned the highest value to 12 out of 33 (36.4%) evaluations for the GPT-3.5 version, and 23 out of 33 (69.7%) for the GPT-4.0 (*p*-value = 0.0067); likewise, Rater 2 assigned the highest value to 18 out of 33 evaluations (54.5%) for the GPT-3.5, and 22 out of 33 (66.7%) for the GPT-4.0. (*p*-value = 0.311).

The mean scores for the three items were 3.36 (±0.72; 84.1%), 3.32 (±0.86; 83%), and 3.57 (±0.89; 89.2%) for correctness, clarity, and exhaustiveness, respectively, without statistically significant differences by the groups (KW *p*-value = 0.78, 0.18 and 0.09, respectively). Inter-observer reliability indicated by Cohen’s Kappa value was 0.52 (*p*-value = 0.0000) for GPT-3.5 and 0.30 (*p*-value = 0.0147) for GPT-4.0.

Both versions of ChatGPT obtained the maximum score for accuracy in answering question number 8. Answers to questions 2 and 7 obtained full marks for version GPT-4.0. Conversely, the answer to question number 11 was completely accurate in version GPT-3.5, the only answer which obtained a higher score than version GPT-4.0. A significant difference in mean scores between the two versions was found by Rater 1 (*p*-value = 0.0107), who indicated that version GPT-4.0 was the most accurate. The answer to question number 3 was graded as completely incorrect by the Raters for both ChatGPT versions.

### 3.2. Qualitative Analysis

Overall, the mean score assigned by the Raters, based on the determinants reported in the Materials and Methods section, to the GPT-4.0 responses was higher than that of the GPT-3.5 responses, with Δ equal to 5.6% for Correctness, 17.9% for Clarity and 9.3% for Exhaustiveness of the answer (Table 3).

In particular, the 11 questions and the evaluations carried out on the basis of the determinants by the two Raters are reported below (S1).

*Q.1*  *Weren’t Diseases Already Disappearing before Vaccines were Introduced Because of Better Hygiene and Sanitation?*

Regarding Clarity of content, both Raters judged the answers offered by the GPT-3.5 version as inaccurate. The imprecise information regarding the transmission route of polio and the reference to the eradication of other vaccine-preventable infectious diseases, apart from smallpox, affected the scoring. Both Raters described the use of more appropriate vocabulary and more complete content as reasons for the higher score given to the Clarity item in the GPT-4.0 version.

*Q.2*  *Which Disease Show the Impact of Vaccines the Best?*

Regarding the response offered by GPT-4.0, the Raters were unanimous in awarding the highest score for all items considered. In contrast, the lack of appropriateness of vocabulary and scientific veracity negatively affected the scores for the Clarity and Correctness items generated by the GPT-3.5 version.

*Q.3*  *What about Hepatitis B? Does That Mean the Vaccine Didn’t Work?*

Overall, the responses given by GPT-3.5 and GPT-4.0 to question Q.3 scored the lowest. In particular, the Raters agreed that the responses from both versions were haphazard from the point of view of the logical description of the content; there were not very exhaustive, and they were difficult to understand. As for the Correctness item, while both Raters considered the information provided by the GPT-4.0 version to be more complete, the inaccuracies in both versions’ responses made the content misleading thereby negatively affecting the score attributed.

*Q.4*  *What Happens if Countries Don’t Immunize against Diseases?*

For both versions of the chatbot, plausibility and scientific veracity positively affected the assigned score, especially in the opinion of Rater 1 for the GPT-4.0 version. On the other hand, the order of the content and the difficulty of comprehension detracted from its Clarity. Finally, for the Exhaustiveness item, the response of the GPT-4.0 version was rated by Rater 2 as less complete than that offered by GPT-3.5.

*Q.5*  *Can Vaccines Cause the Disease? I’ve Heard That the Majority of People Who Get Disease Have Been Vaccinated.*

In the GPT-3.5 version, the logical order negatively affected the Clarity of the response for both Raters. In contrast, scientific veracity for Rater 1 and degree of comprehensiveness of the response for Rater 2 were the determinants that accounted for the highest score awarded to Correctness and Exhaustiveness, respectively. In the GPT-4.0 version, for both Raters, ease of comprehension and logical order positively affected the scoring, while imprecision regarding HBV and HPV vaccine definitions negatively affected the rating given for Correctness according to Rater 1.

*Q.6*  *Will Vaccines Cause Harmful Side Effects, Illnesses or Even Death? Could There Be Long Term Effects We Don’t Know about Yet?*

The GPT-4.0 version was considered more correct, clear, and exhaustive than the GPT-3.5 version. Specifically, with regard to Correctness, the Raters considered both responses to be sufficiently plausible, but the lack of appropriate references to pharmacovigilance accounted for the lower score in the response provided by the GPT-3.5 version.

*Q.7*  *Is it True That There Is a Link between the Diphtheria-Tetanus-Pertussis (DTP) Vaccine and Sudden Infant Death Syndrome (SIDS)?*

The Raters agreed that the answers provided by the chatbots were sufficiently plausible and evidence-based. This resulted in the highest score being given to the Correctness item. However, the level of comprehension and appropriateness of vocabulary allowed a higher score to be assigned to the Clarity of the response of GPT-4.0 than to GPT-3.5. In addition, Rater 1 considered the GPT-4.0 version more complete than GPT-3.5.

*Q.8*  *Isn’t Even a Small Risk Too Much to Justify Vaccination?*

For both versions of the chatbot, the Raters considered Correctness, Clarity, and Exhaustiveness of the answers to be no less accurate than those of the answers provided by WHO, assigning the highest score to all items.

*Q.9*  *Vaccine-Preventable Diseases Have Been Virtually Eliminated from My Country. Why Should I Still Vaccinate My Child?*

The Raters agreed in assigning the highest score to the response provided by GPT-3.5 considering the contents to be correct, clear, and exhaustive. According to Rater 2, some of the content of the response provided by GPT-4.0 was considered inaccurate, particularly in the definition of the concept of herd immunity, resulting in a lower score being assigned to the Correctness item.

*Q.10*  *Is It True That Giving a Child Multiple Vaccinations for Different Diseases at the Same Time Increases the Risk of Harmful Side Effects and Can Overload the Immune System?*

Rater 1 considered the GPT-4.0 version more correct, clear, and exhaustive than the GPT-3.5 version, as the closure provided in the latter penalized the consistency, logical order, and degree of completeness of the response. In contrast, the responses of both versions were considered equivalent by Rater 2, although the inaccuracy in reference to the co-administration of vaccines negatively affected the assessment of Correctness.

*Q.11*  *Why Are Some Vaccines Grouped Together, Such as Those for Measles, Mumps and Rubella?*

For both Raters, the GPT-3.5 version was the most correct, clear, and exhaustive for the entire set of responses. In contrast, serious content errors were found in the GPT-4.0 version in relation to potential negative interactions among combined vaccines.

## 4. Discussion

The emergence of innovative and advanced LLMs such as ChatGPT has given rise to a range of concerns and debates, and as such, it is crucial to discuss its potential benefits, future perspectives, and limitations [40,41]. On the one hand, such LLMs could constitute a revolutionary change in education as a whole, as well as in research and academic writing [42,43]. On the other hand, the same technology could facilitate the spread of misinformation and of other types of information detrimental to users, especially in the field of health topics [44,45].

In the present study, we examined the Correctness, Clarity, and Exhaustiveness of ChatGPT responses to common vaccination myths and misconceptions similarly to what WHO did with its responses. Overall, the Raters perceived that the ChatGPT findings provided accurate and comprehensive information on common myths and misconceptions about vaccination in an easy-to-understand, conversational manner, without providing misinformation or harmful information. In particular, the determinants that had the greatest impact on the scores assigned were: scientific veracity, appropriateness of vocabulary, and the logical order chosen for the description of the contents with regard to Clarity and to the completeness of the answer for the Exhaustiveness item.

Nevertheless, in some cases, several aspects of the description of the contents could be improved. For example, in the Raters’ opinion, the answers given by both versions of ChatGPT to Question 2 were misleading. In particular, citing immunization against smallpox as the only example of the significant impact of vaccination, the chatbot suggested that the eradication of the disease they prevent is the only tangible benefit. From ChatGPT, it is not clear why the implementation of mass vaccination is not directly followed by a dramatic drop in the disease incidence. Indeed, the AI tool appears to entirely disregard the benefits offered by vaccination in the short term (e.g., the management of infection clusters and management of the disease as demonstrated with the COVID-19 vaccination) and in the long term (e.g., the impact of vaccination on economic growth and on the sustainability and efficiency of health systems) [46,47,48]. 

This is worrying if one considers that nowadays “convenience” and “complacency” are among the main determinants of vaccine hesitancy and any perception that the vaccine may not be essential in the prevention of infectious diseases may discourage citizens from adhering to vaccination programs [49,50,51,52]. Indeed, alongside advanced technologies, accurate and accessible medical information communicated by public health operators, particularly in a context of low health literacy, is essential to providing patients with the information needed to improve their understanding and enable them to make informed decisions about their care [53,54,55,56]. It should be noted that the same chatbot advises, both in the answer to Question 3 and Question 6, that it is important to consult your doctor to discuss any concerns or specific circumstances that could influence your decision to be vaccinated.

Moreover, regarding the Correctness of the answers provided, the Raters identified numerous inaccuracies for both versions. In particular, errors regarding the transmission route and the eradication circumstances of some infectious diseases (Question 1) were found. Misclassifications of the HBV (Hepatitis B Vaccine) and HPV (Human Papilloma Virus Vaccine) vaccines, cited as examples of live attenuated vaccines, were noted in the response to Question 5. Other serious inaccuracies were found in the answers to Question 10 and Question 11. In particular, in Q.10, there are clear references only to combined vaccines, with no mention of the rare cases in which the co-administration of vaccines is expressly contraindicated. In the Raters’ opinion, this limits the transparency of the answer and could cause the user to suspect a potential cover-up of the albeit limited contraindications to the co-administration of vaccines, which are expressly reported in the Summary of Product Characteristics (SPC) as for any other drug [57]. Similarly, in Q.11, it is asserted that “combining vaccines can reduce the likelihood of side effects and the potential for negative interactions between vaccines” without mentioning that the combination of several vaccines can sometimes increase reactogenicity (as in the case of the MMRV vaccine, with side effects such as febrile seizures). This concept should also have been expressed more clearly by mentioning that the administration of separate doses can lead to repeated occasions of local events, also described in each SPC [58].

A separate consideration must be made for the answer to Question 3, which received a considerably lower score than the others, causing the authors to suspect that the question may not have been asked correctly. In this regard, the literature describes how even in the common administration of a survey, the consequentiality of the questions could influence the answers given. In fact, even in the WHO questions, Question 3 seems to follow on from the previous one. Therefore, since ChatGPT remembers previous interactions within the same conversation, we decided to resubmit the two questions to both versions of ChatGPT consecutively (within the same conversation) as opposed to independently. In this case, albeit with further room for improvement in terms of Clarity and Exhaustiveness, the Raters deemed the answers returned by GPT-3.5 and GPT-4.0 to have improved significantly, highlighting the fact that the tool may have misunderstood the original question or did not have sufficient elements to generate a completely exhaustive answer. This could stem from the fact that some answers to topics which are as widely debated and rich in history as vaccinations not only assume an in-depth knowledge but also imply that this very knowledge gives rise to a reasoning which is then applied [59,60].

The above-mentioned is relevant when one considers that people are often unaware how accurate and personalized information is obtained and tend to implicitly trust something that mimics human behaviors and responses, such as AI. They therefore fail to validate the information which, when conveyed by tools as up-to-date and widely discussed by the virtual community as ChatGPT, is deemed to be accurate and reliable [61,62,63].

All things considered, given that ChatGPT is expected to improve significantly in very little time, thanks to the continuous updating and refinement of the algorithms and model parameters, the quality and reproducibility of the responses are likely to improve. On the other hand, the fact that only one of the two Raters found a significant difference between the two versions implies that even experts may have differing opinions when answering these questions.

In this regard, many studies in the literature describe how the interpretation of a concept is not only the result of scientific knowledge but also the product of the coordinated actions of various processes such as perception, attention, imagination, thought and memory, which, when added to knowledge, contribute to the elaboration of the perceived concept [64,65,66]. Thus, it follows that a lack of the basic knowledge necessary to discern between what is correct, clear, and exhaustive versus what is not, must be taken into account when referring to how the general public can question an AI whose aseptic and decontextualized responses can influence the reader’s interpretation of the content.

This means that the use of these tools in healthcare settings will require careful consideration in order to prevent potentially detrimental uses, such as bypassing professional medical advice and ethical issues, including the potential risk of bias and factual inaccuracies [67,68]. This was clearly seen during the COVID-19 pandemic, where the spread of misinformation resulted in a growing infodemic [69,70]. In fact, in a context of continuous media exposure to an enormous volume of apparently conflicting news for an inexperienced user, as well as the conflicting opinions on the efficacy of the different vaccines available, finding reliable and safe sources of information was described as a major source of uncertainty [71,72].

Additionally, since these AI tools are only as trustworthy as the data they are trained on, it is important to consider privacy and ethical issues as well. Indeed, the fact that the system does not clarify the sources from which it draws the information could certainly constitute a problem, especially for those aiming to address or investigate scientific issues. Furthermore, many scientific models contain “black boxes”, simplified constructs that omit or completely ignore the details of the underlying mechanisms, constituting a serious methodological problem in the scientific field and highlighting the existence of an approach to science focused solely on explanation and/or simplification. However, ChatGPT’s own answers underlined the importance of reliable and in-depth sources of information, as well as the use of terms associated with uncertainty, emphasizing that the results generated are no substitute for clinical consultation of healthcare professionals.

Finally, the fact that ChatGPT is available for free allows even the most economically disadvantaged patients to access reliable and personalized medical information. On the other hand, the availability of a better-performing version (GPT-4.0) only for paying users, poses the problem of equality in accessing information. Even if we take into consideration the fact that although ChatGPT-3.5 is free, many cannot access it for economic or cultural reasons and are therefore excluded from these sources of information [73,74].

Overall, ChatGPT, and AI tools in general, has the potential to be a valuable resource both for providing immediate medical information to patients and for improving healthcare efficiency and decision-making for healthcare professionals. Indeed, if evaluated and trained by experts on controlled medical information, LLMs like ChatGPT could rapidly transform the communication of medical knowledge.

### Study Limitations

The results of the present study should be evaluated based on the following limitations. First, given that the general body of text data ChatGPT is trained on dates back to 2021, accuracy could be scientifically outdated for some topics. However, the WHO published their myths and misconceptions about vaccinations in late 2020, so the information available for compiling answers overlapped. Second, this study was based on a subjective assessment of the content, and this approach may produce slightly varying results based on the expertise of individual evaluators. Moreover, the Raters knew which version the responses were from, so their rating may have been influenced by the pre-conception of higher capacity of GPT-4.0 versus GPT-3.5. However, it is essential to take into consideration the very high-level of professionalism of the experts involved, as well as their skills in the field of vaccination communication, and the fact that the primary objective of the study was not specifically to make a comparison between the two versions but to verify, also in consideration of the fact that the more advanced version is paid and therefore less accessible to many users, whether both versions could provide information suitable for users. In fact, in one question, ChatGPT-3.5 received a higher score than the more advanced version. In any case, as stated on the ChatGPT landing page, it may occasionally produce malicious instructions or biased content, especially considering that the quality and accuracy of the dataset used to train the tool are unknown.

## 5. Conclusions

LLM technologies, including ChatGPT, represent a further incremental step, and they are rapidly becoming more widespread, generating both opportunities and concerns regarding their potential misuse. Considering their wide availability and potential societal impact, it is critical to exercise caution, acknowledge their limitations and develop appropriate guidelines and regulations with the involvement of all the relevant stakeholders. In particular, the quality of this innovative approach depends, and will continue to depend, more and more on the ability to ask the correct questions as well as on the critical ability of those who use it and will use it, as possible ethical and legal issues could limit potential future applications.

If implemented correctly, ChatGPT could have a transformative impact both in research, by making it more automated or simplified, and in healthcare, by augmenting rather than replacing human expertise, thus ultimately improving the quality of life for many patients. However, despite displaying a high level of Correctness, Clarity, and Exhaustiveness, further studies are needed to improve the reliability of these tools in the online communication environment, particularly concerning patient education, and to ensure their safe and effective use before clinical integration.

## Figures and Tables

**Table 1:** WHO’s list of eleven myths and misconceptions * relating to vaccines and immunization.

Weren’t diseases already disappearing before vaccines were introduced because of better hygiene and sanitation? Which disease show the impact of vaccines the best? What about hepatitis B? Does that mean the vaccine didn’t work? What happens if countries don’t immunize against diseases? Can vaccines cause the disease? I’ve heard that the majority of people who get disease have been vaccinated. Will vaccines cause harmful side effects, illnesses or even death? Could there be long term effects we don’t know about yet? Is it true that there is a link between the diphtheria-tetanus-pertussis (DTP) vaccine and sudden infant death syndrome (SIDS)? Isn’t even a small risk too much to justify vaccination? Vaccine-preventable diseases have been virtually eliminated from my country. Why should I still vaccinate my child? Is it true that giving a child multiple vaccinations for different diseases at the same time increases the risk of harmful side effects and can overload the immune system? Why are some vaccines grouped together, such as those for measles, mumps and rubella?

* The questions were worded exactly as given on the WHO website, notwithstanding the typo in Question 2.

**Table 2:** Scores and mean values of accuracy assigned by Raters to the answers provided by GPT-3.5 and GPT-4.0.

Q	Items	Groups				Item Mean	Mean 3.5	Mean 4.0	Total Mean
		Rater 1		Rater 2					
		GPT-3.5	GPT-4.0	GPT-3.5	GPT-4.0				
1	Correctness	3	3	3	4	3.25	2.67	3.50	3.08
	Clarity	2	3	2	3	2.50			
	Exhaustiveness	3	4	3	4	3.50			
2	Correctness	3	4	3	4	3.50	3.17	4.00	3.58
	Clarity	3	4	2	4	3.25			
	Exhaustiveness	4	4	4	4	4.00			
3	Correctness	2	3	1	2	2.00	1.17	2.17	1.67
	Clarity	1	2	1	2	1.50			
	Exhaustiveness	1	2	1	2	1.50			
4	Correctness	3	4	3	3	3.25	3.17	3.33	3.25
	Clarity	3	3	3	3	3.00			
	Exhaustiveness	3	4	4	3	3.50			
5	Correctness	4	3	3	4	3.50	3.33	3.83	3.58
	Clarity	3	4	3	4	3.50			
	Exhaustiveness	3	4	4	4	3.75			
6	Correctness	3	4	3	3	3.25	3.33	3.83	3.58
	Clarity	3	4	4	4	3.75			
	Exhaustiveness	3	4	4	4	3.75			
7	Correctness	4	4	4	4	4.00	3.50	4.00	3.75
	Clarity	3	4	3	4	3.50			
	Exhaustiveness	3	4	4	4	3.75			
8	Correctness	4	4	4	4	4.00	4.00	4.00	4.00
	Clarity	4	4	4	4	4.00			
	Exhaustiveness	4	4	4	4	4.00			
9	Correctness	4	4	4	3	3.75	4.00	3.83	3.92
	Clarity	4	4	4	4	4.00			
	Exhaustiveness	4	4	4	4	4.00			
10	Correctness	3	4	3	3	3.25	3.33	3.83	3.58
	Clarity	3	4	4	4	3.75			
	Exhaustiveness	3	4	4	4	3.75			
11	Correctness	4	2	4	3	3.25	4.00	3.17	3.58
	Clarity	4	3	4	4	3.75			
	Exhaustiveness	4	3	4	4	3.75			
Total items’ mean	Correctness	3.36	3.55	3.18	3.36	3.36			
	Clarity	3.00	3.55	3.09	3.64	3.32			
	Exhaustiveness	3.18	3.73	3.64	3.73	3.57			
Total	mean	3.18	3.61	3.30	3.58	3.42	3.24	3.59	3.42
	DS	0.80	0.65	0.94	0.65	0.65	0.87	0.65	0.79
	%	79.5	90.2	82.6	89.4	85.4	81.1	89.8	85.4

**Table 3:** Mean values of the three items assigned by Raters on the answers provided by GPT-3.5 and GPT-4.0.

Item	Mean Score		Percentage (%)		Δ (%)
	GPT-3.5	GPT-4.0	GPT-3.5	GPT-4.0	
**Correctness**	3.27	3.45	81.8	86.4	5.6
**Clarity**	3.05	3.59	76.1	89.8	17.9
**Exhaustiveness**	3.41	3.73	85.2	93.2	9.3

Correctness: plausibility, coherence, scientific veracity, and evidence; Clarity: ease of understanding, appropriateness of vocabulary, conciseness, and logical order; Exhaustiveness: degree of completeness.

## Data Availability

Data are available on reasonable request.
